# Biotechnological approaches for the production of camptothecin

**DOI:** 10.1007/s00253-024-13187-2

**Published:** 2024-06-19

**Authors:** Akshatha Banadka, Sudheer Wudali Narasimha, Vijayalaxmi S. Dandin, Poornanand M. Naik, Amaranatha Reddy Vennapusa, Kalpalatha Melmaiee, Ramu S. Vemanna, Jameel M. Al-Khayri, Muthu Thiruvengadam, Praveen Nagella

**Affiliations:** 1 Department of Life Sciences, CHRIST (Deemed to be University), Bangalore, 560 029 Karnataka India; 2 Department of Biology, JSS College, Dharwad, India; 3 Department of Botany, Karnatak University, Dharwad, 580003 Karnataka India; 4 Department of Agriculture and Natural Resources, Delaware State University, Dover, DE 19901 USA; 5 Laboratory of Plant Functional Genomics, Regional Center for Biotechnology, Faridabad, 121001 Haryana India; 6 Department of Agricultural Biotechnology, College of Agriculture and Food Sciences, King Faisal University, Al- Ahsa, 31982 Saudi Arabia; 7 Department of Crop Science, College of Sanghuh Life Science, Konkuk University, Seoul, South Korea

**Keywords:** Camptothecin, *In vitro* production, Camptotheca acuminata, Extraction, Quantification, *NothapodytesNimmoniana*

## Abstract

**Abstract:**

Camptothecin (CPT), an indole alkaloid popular for its anticancer property, is considered the third most promising drug after taxol and famous alkaloids from *Vinca* for the treatment of cancer in humans. Camptothecin was first identified in *Camptotheca acuminata* followed by several other plant species and endophytic fungi. Increased harvesting driven by rising global demand is depleting the availability of elite plant genotypes, such as *Camptotheca acuminata* and *Nothapodytes nimmoniana*, crucial for producing alkaloids used in treating diseases like cancer. Conservation of these genotypes for the future is imperative. Therefore, research on different plant tissue culture techniques such as cell suspension culture, hairy roots, adventitious root culture, elicitation strategies, and endophytic fungi has been adopted for the production of CPT to meet the increasing demand without affecting the source plant’s existence. Currently, another strategy to increase camptothecin yield by genetic manipulation is underway. The present review discusses the plants and endophytes that are employed for camptothecin production and throws light on the plant tissue culture techniques for the regeneration of plants, callus culture, and selection of cell lines for the highest camptothecin production. The review further explains the simple, accurate, and cost-effective extraction and quantification methods. There is enormous potential for the sustainable production of CPT which could be met by culturing of suitable endophytes or plant cell or organ culture in a bioreactor scale production. Also, different gene editing tools provide opportunities for engineering the biosynthetic pathway of CPT, and the overall CPT production can be improved .

**Key points:**

• *Camptothecin is a naturally occurring alkaloid with potent anticancer properties, primarily known for its ability to inhibit DNA topoisomerase I.*

• *Plants and endophytes offer a potential approach for camptothecin production.*

• *Biotechnology approaches like plant tissue culture techniques enhanced camptothecin production.*

**Supplementary information:**

The online version contains supplementary material available at 10.1007/s00253-024-13187-2.

## Introduction

**Fig. 1:**
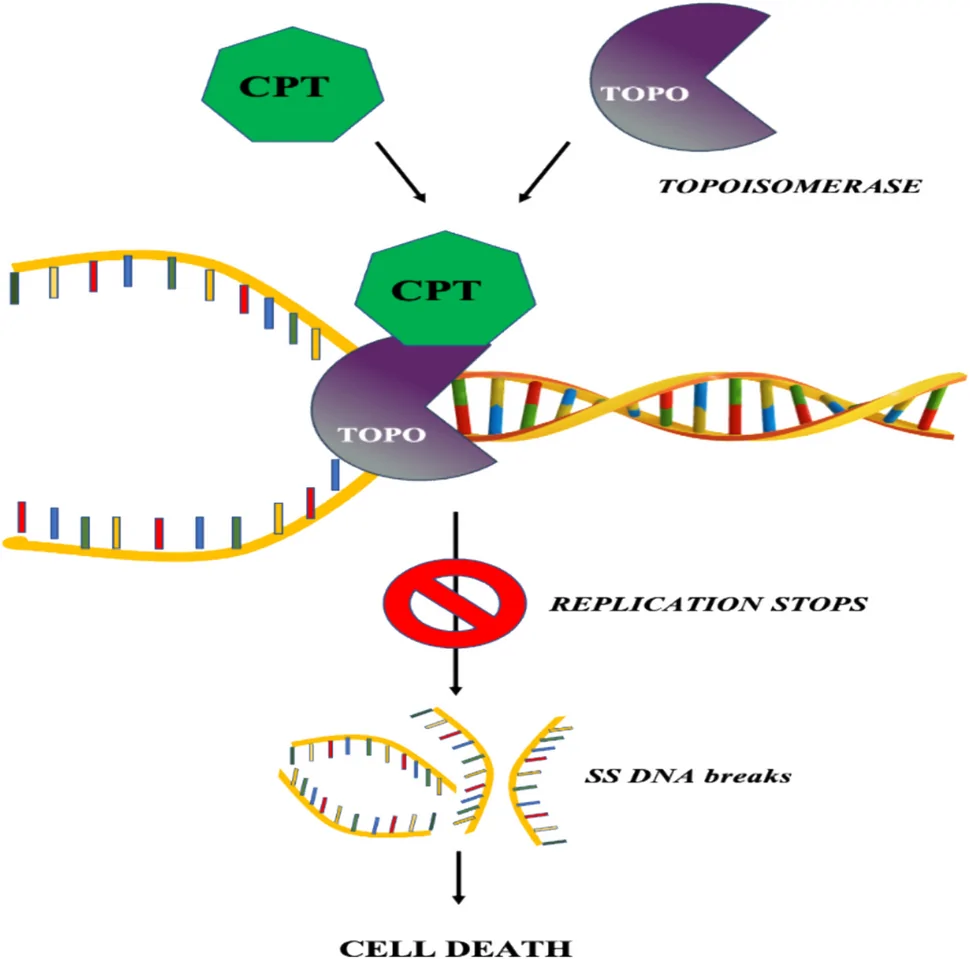
Mechanism of inhibition of Topoisomerase I activity by camptothecin

Camptothecin (CPT) is a monoterpene indole alkaloid produced by plants as a secondary metabolite (Sadre et al. 2016). CPT has been a propitious chemotherapeutic drug since its discovery in 1966 by Wall and Wani (Malik and Laura 2014). CPT is used in combination with other chemotherapeutic drugs to enhance anti-cancerous properties with the further advantage of lesser side effects when compared to invasive chemotherapy (Nurgali et al. 2018). The drug is used for the treatment of metastatic cancer in organs such as the lung, breast, gastrointestinal tract, liver, gallbladder, spleen, and colon (Kamble et al. 2011). The drug exhibits its anticancer property by inhibiting the DNA topoisomerase I enzyme. DNA topoisomerase enzyme plays a predominant role in DNA replication, transcription, repair, and recombination processes (Ulukan and Swaan 2002). It is the third most promising anti-cancer drugs of the twenty-first century after taxol and vinca alkaloids (Mohinudeen et al. 2021). Figure 1 illustrates the inhibition of topoisomerase I activity by camptothecin.

CPT was first discovered in the Chinese deciduous tree, *Camptotheca acuminata* Decne. Later, the alkaloid was reported to be distributed among plant species of various families such as *Nyssaceae*, *Icacinaceae*, *Loganiaceae*, *Apocynaceae*, and *Rubiaceae* and their presence in endophytes such as *Entrophospora infrequens*,* Fusarium solani*, and *Neurospora* (Pu et al. 2019). The increasing market demand for CPT from these herbal plants has resulted in overharvesting and affected the existence of nativity (Niazian 2019; Greenwell and Rahman 2015). Thus, establishing alternative strategies for camptothecin production using biotechnological approaches provides a viable option. Plant tissue culture offers an alternative method for the conservation of the species. For the enhanced CPT production, different plant tissue culture techniques such as micropropagation, indirect and direct organogenesis, and hairy root culture have been employed from these plants (Malik and Laura 2014). Additionally, an effective strategy of genetic manipulation has been employed recently in enhancing CPT yield (Kai et al. 2015). Moreover, improved extraction methods have helped in isolating the compound efficiently. A simple, accurate, and cost-effective quantification method was developed for the quantification of CPT (Lokesh et al. 2014).

A good number of studies on camptothecin-producing plants and endophytes have been reported. However, there is no extensive documentation on various plant tissue culture methods and optimization strategies for enhanced camptothecin production from different plants and endophytes. There are no substantial reports covering the different extraction and quantification methods of camptothecin and its large-scale production. So, in view of this, the present review provides detailed information on the structure and function of a highly effective anticancer drug, CPT. It reports the plants and endophytes that are utilized for CPT production and discusses the alternative methods for CPT production over conventional methods, which are robust and cost-effective. The alternative approaches include callus, cell suspension, shoot, and root cultures (adventitious root and hairy root). Further, the review discusses the optimization strategies used to enhance the camptothecin production with optimization of media and culture conditions. Elaborative details on the use of bioreactor for the large-scale production of CPT and summary on the current status of metabolic engineering of CPT biosynthetic pathways have been discussed.

## Camptothecin: structure and its derivatives/analogues

**Fig. 2:**
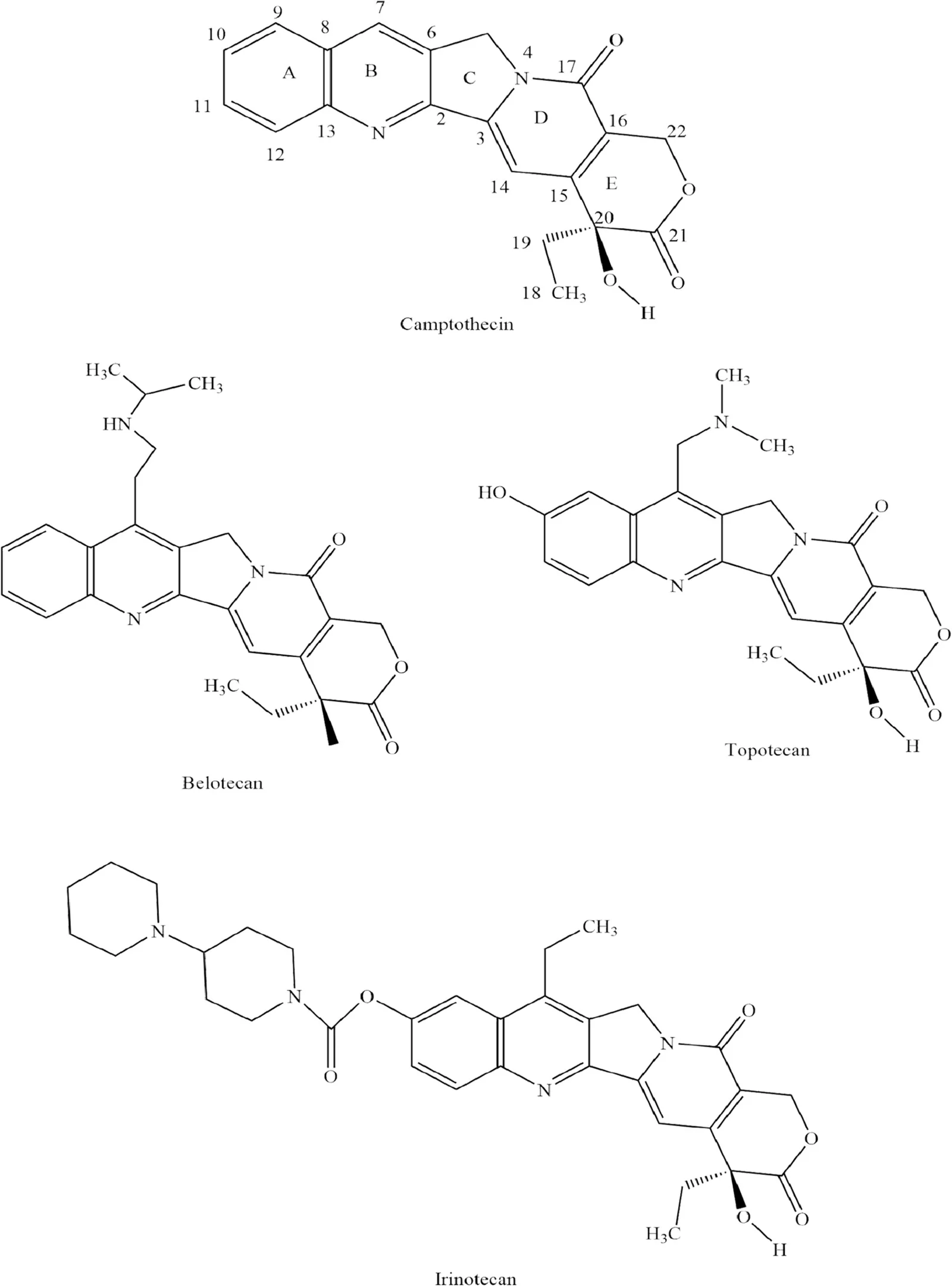
Structure of camptothecin and its derivatives/analogues (Martino et al. 2017)

CPT is a pentacyclic alkaloid that was first discovered in the early 1960s (Malik and Laura 2014). The CPT structure comprises a pyrrolo (3,4-β) quinoline moiety, a conjugated pyridone, and an asymmetric center at the 20th position within the α-hydroxy lactone ring with 20 (S) configuration. The pentacyclic ring system consisting of A, B, C, D, and E rings majorly contributes to the topoisomerase inhibition activity of CPT (Kamble et al. 2011). Since its structural elucidation in 1966, CPT has undergone evolution through structural modifications and several CPT derivatives have been generated. These analogues were obtained based on the structural-activity relationships (SAR) which include Topotecan, Irinotecan, Etirinotecan, pegol, Belotecan, Rubitecan, Diflomotecan, Lurtotecan, and Exatecan. Currently, three water soluble anticancer analogues of CPT, Irinotecan, Topotecan, and Belotecan have been approved and are commercially available for cancer treatment (Li et al. 2017). Figure 2 illustrates the structure of camptothecin and its derivatives.

### Topotecan

9-Dimethylaminomethyl-10-hydroxycamptothecin (Topotecan) was developed by the National Cancer Institute in collaboration with the University of Florida at Gainesville and SmithKline Beecham headed by Dr. Warren Ross in the 1980s. It was first approved by FDA in 1996 and is now manufactured by GlaxoSmithKline and commercially available as Hycamtin (developmental therapeutics program). It is synthesized from 10-hydroxy-20-(S)-camptothecin or with dimethylamine by aminomethylation in the presence of catalyst trihalomethane (Puri et al. 2003). Topotecan is used in the treatment of ovarian cancer, small cell and non-small cell lung cancers, non-Hodgkin lymphoma, endometrial cancer, oligodendroglioma, and breast cancer.

### Irinotecan

7-Ethyl-10-[4-(1-piperidino)-1-piperidino] carbonyl oxy-camptothecin (Irinotecan), commonly available under the brand name Camptosar, is prepared by ethylating 10-[4-(1-piperidino)-1-piperidino] carbonyloxy-camptothecin at the 7th position (Zabudkin 2015). Irinotecan was first developed in 1983 by Yakult Honsha Co., Ltd., Japan. In 1994, it was first approved in Japan for its use (Fukuoka 2001). Irinotecan is used in the treatment of small-cell lung cancer, colon cancer, ovarian cancer, acute and lymphoblastic leukemia, and non-Hodgkin’s lymphoma (Kamble et al. 2011).

### Belotecan

(20 S)-7-(2-isopropylamino)-ethyl-camptothecin commercially available as Camtobell marketed by Chong Kun Dang Corp. (Seoul, Korea) was first prepared by Ahn and coworkers of Chong Kun Dang in 1999 (Ahn et al. 2000). It has DNA topoisomerase inhibition activity in cancer cells. Belotecan is synthesized in a two-step reaction: Minisci type reaction, which involves conversion of CPT 7-methylcamptothecin, and Mannich type reaction, which involves conversion of 7-methyl CPT to belotecan (Liew and Yang 2008). Belotecan is used in the treatment of small-cell lung cancer and ovarian cancer (Liew and Yang 2008).

### Camptothecin distribution in plants

Earlier, CPT was limited to only two plants: *C. acuminata* of the *Nyssaceae* family and *Nothapodytes nimmoniana* from the *Icacinaceae* family. However, the increased CPT demand led to several research studies to identify alternative CPT-producing plants. At present, CPT is found to be distributed among plant species of various plant families such as *Nyssaceae*, *Icacinaceae*, *Loganiaceae*, *Apocynaceae*, and *Rubiaceae* (Ulukan and Swaan 2002). The seeds of *C. acuminata* and *N. nimmoniana* have reported the presence of 0.110% CPT (Liu and Adams 1996) and 0.179% CPT (Isah and Mujib 2015a), respectively. Some of the *Ophiorrhiza* species such as *O. fucosa*, *O. plumbea*, *O. ridleyana, *and *O. harrisiana* have reported the presence of camptothecin in them (Viraporn et al. 2011). The highest CPT content of 1.418% has been reported in the seeds of *Miquelia dentata* Bedd. (Ramesha et al. 2013). For the production of one ton of camptothecin, nearly 1000–1500 tons of plant material is required. The annual marketing sales of camptothecin and its derivatives have been estimated to be $1 billion (Shrivastava et al. 2021). Table 1 presents the parts of the various plant species from which CPT has been isolated.

**Table 1:** Camptothecin production in different parts of the plant species

Plant Name	Family	Site of production	Content of CPT	Reference
*Camptotheca acuminata*	*Nyssaceae*	Shoot	0.042%	(Liu and Adams 1996)
		Root	0.051%	
		Leaf	0.015%	
		Branch	0.062%	
		Seed	0.110%	
*Camptotheca lowreyana* S.Y.Li	*Nyssaceae*	Young leaves	0.5537%	(Li et al. 2002)
		Old leaves	0.118%	
*Camptotheca yunnanensis* Dode	*Nyssaceae*	Young leaves	0.4494%	
		Old leaves	0.059%	
*Chonemorpha grandiflora* G.Don	*Apocynaceae*	Stem bark	0.0013%	(Kulkarni et al. 2010)
		Leaves	0.0009%	
*Nothapodytes nimmoniana*	*Icacinaceae*	Shoots	0.075–0.81%	(Isah and Mujib 2015a)
		Root	0.11–0.2%	
		Seeds	0.179%	
		Leaves	0.081-0.7%	
		Fruits	0.122%	
*Mappia pittosporoides* Oliv.	*Icacinaceae*	Leaves	0.238%	(Zeng et al. 2013)
		Fruits	0.102%	
		Roots	0.172%	
*Ervatamia heyneana* (Wall.) T.Cooke (Syn:*Tabernaemontana alternifolia* L.)	*Apocynaceae*	Stem bark	0.00013%	(Gunasekera et al. 1979)
			0.0003%	(Kulkarni 2008)
		Leaves	0.0001%	
*Merrilliodendron megacarpum* (Hemsl.) Sleumer	*Icacinaceae*	Stem bark	0.053%	(Arisawa et al. 1981)
*M. dentata*	*Icacinaceae*	Leaf	0.024%	(Ramesha et al. 2013)
		Cotyledon	1.418%	
		Root	0.153%	
		Fruit	1.22%	
		Twig	0.003%	
*Mostuea brunonis* Didr.	*Loganiaceae*	Whole plant	0.01%	(Dai et al. 1999)
*Pyrenacantha klaineana* Pierre ex Exell & Mendonça	*Icacinaceae*	Stem	0.00048%	(Zhou et al. 2000)
		Fruit	0.488%	(Ramesha et al. 2013)
*Ixora coccinea* L.	*Rubiaceae*	Young leaves	0.4146 µg g^−1^	(Saravanan and Boopalan 2011)
		Mature leaves	5.0611 µg g^−1^	
*Ophiorrhiza. alata* Craib	*Rubiaceae*	Leaves	83 µg g^−1^	(Krishnakumar et al. 2020)
		Root	388 µg g^−1^	
*Ophiorrhiza rugosa* var. *decumbens* (Gardner ex Thwaites) Deb & Mondal	*Rubiaceae*	Whole plant	4.20 µg g^−1^	(Krishnakumar et al. 2020)
		Shoot	2 µg g^−1^	
		Root	24 µg g^−1^	
*Ophiorrhiza rugosa* var. *prostrata* (D.Don) Deb & Mondal	*Rubiaceae*	Stem	0.08%	(Gharpure et al. 2010)
		Root	0.16%	
		Fruit	0.0165%	
		Young leaves	0.0062%	
		Mature leaves	0.0022%	
*Ophiorrhiza filistipula* Miq.	*Rubiaceae*	Leaves	0.00009%	(Arbain et al. 1993)
*Ophiorrhiza mungos* L.	*Rubiaceae*	Root	0.0176%	(Roja 2006)
		Shoot	0.0096%	
		Young leaves	1664 µg g^−1^	(Wetterauer et al. 2021)
		Mature leaves	2000 µg g^−1^	
*Ophiorrhiza mungos* L. var. *angustifolia* (Thw.) Hook. f.	*Rubiaceae*	Whole plant	297.94 µg g^−1^	(Krishna Kumar et al. 2018)
			127.86–476.89 µg g^−1^	(Rajan et al. 2013)
*Ophiorrhiza grandiflora* Wight	*Rubiaceae*	Whole plant	1.07–1.34 µg g^−1^	(Rajan et al. 2013)
*Ophiorrhiza shendurunii* A.E.S.Khan, E.S.S.Kumar & Pushp.	*Rubiaceae*	Whole plant	0.05 µg g^−1^	(Rajan et al. 2013)
*Ophiorrhiza pectinata* Arn.	*Rubiaceae*	Whole plant	0.28–38.65 µg g^−1^	(Rajan et al. 2013)
*Ophiorrhiza trichocarpa* Blume	*Rubiaceae*	Whole plant	19.50–28.31 µg g^−1^	(Rajan et al. 2013)
*Ophiorrhiza pumila*	*Rubiaceae*	Whole plant	0.0300–0.0510%	(Saito et al. 2001)
		Leaves	0.03–0.04%	
		Root	0.10%	
*Ophiorrhiza japonica* Blume	*Rubiaceae*	Whole plant	0.0073%	(Guo-yin 2009)

### Camptothecin distribution in the endophytes

Endophytes are the microorganisms like bacteria or fungi that coexist with a living plant and are reported to be the source for antibiotics, antiviral compounds, anti-diabetic agents, anticancer agents, and many more. In recent times, they serve as an alternative source to produce plant secondary metabolites such as taxol, camptothecin, capsaicin, rohitukine, and several other such compounds (Uzma et al. 2018). The fungal species of *Aspergillus*, *Trichoderma*, *Fomitopsis. Phomposis, *and *Fusarium* have been reported to produce CPT (Malik and Laura 2014). Three CPT-producing fungi *T atroviridae* LY357, *Aspergillus* sp. LY341, and *Aspergillus* sp. LY355 isolated from barks, twigs, leaves, and fruits of *C. acuminata*. were 7.93, 42.92, and 197.82 µg L^−1^, respectively (Pu et al. 2013). *F. oxysporum kolhapuriensis* from the *Nectriaceae* family reported the presence of 283 mg L^−1^ CPT (Bhalkar et al. 2015). Table 2 presents the production of CPT from different endophytes.

**Table 2:** Production of camptothecin from some of the endophytes

Endophyte name	Family	Content of CPT	Reference
*Trichoderma atroviridae LY357*	*Hypocreaceae*	197.82 µg L^−1^	(Pu et al. 2013)
*Aspergillus sp. LY341*	*Trichocomaceae*	7.93 µg L^−1^	(Pu et al. 2013)
*Aspergillus sp LY355*	*Trichocomaceae*	42.92 µg L^−1^	(Pu et al. 2013)
*Fusarium solani MTCC 9667*	*Nectriaceae*	37 µg 100 g^−1^	(Shweta et al. 2010)
*Fusarium solani MTCC 9668*	*Nectriaceae*	53 µg 100 g^−1^	(Shweta et al. 2010)
*Fusarium oxysporum kolhapuriensis*	*Nectriaceae*	283 mg L^−1^	(Bhalkar et al. 2015)
*Alternaria alstroemeriae*	*Pleosporaceae*	426.7 µg g^−1^	(Mohinudeen et al. 2021)
*Alternaria burnsii*	*Pleosporaceae*	403.3 µg g^−1^	(Mohinudeen et al. 2021)
*Anthracnose fungus SUK1 (F1)*	*Glomerellaceae*	69 mg L^−1^	(Mohinudeen et al. 2021)
*Corynespora cassiicola*	*Corynesporascaceae*	146 mg L^−1^	(Mohinudeen et al. 2021)
*Entrophospora infrequens*	*Acaulosporaceae*	4.96 mg 100 g^−1^	(Murthy et al. 2019)
*Fusarium. oxysporum NFX06*	*Nectriaceae*	610.09 ng g^−1^	(Musavi et al. 2015)
*Neurospora crassa*	*Sordariaceae*	5.5 µg g^−1^	(Rehman et al. 2008)
Nodulisporium	*Xylariaceae*	5.5 µg g^−1^	(Rehman et al. 2008)
*Fomitopsis* sp. (MTCC 10,177)	*Fomitopsidaceae*	55.49 µg g^−1^	(Shweta et al. 2013)
*Phomopsis* sp	*Valsaceae*	42.06 µg g^−1^	(Shweta et al. 2013)
*Alternaria alternata* (MTCC 5477)	*Pleosporaceae*	73.9 µg g^−1^	(Shweta et al. 2013)

### Biosynthetic pathway of camptothecin

**Fig. 3:**
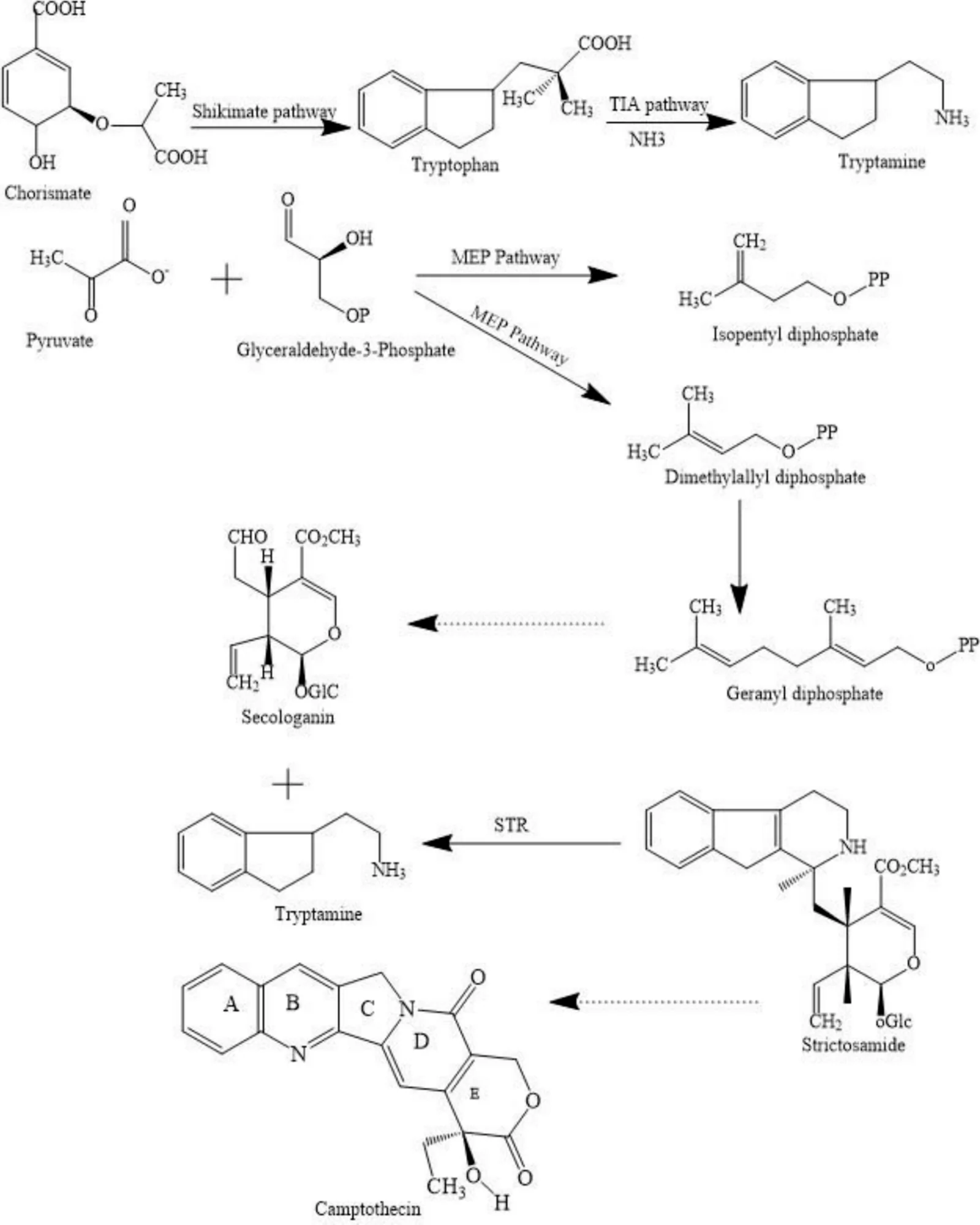
The biosynthetic pathway of camptothecin involves the pre-strictosidine, strictosidine synthesis, and post-strictosidine pathways. TIA, monoterpenoid indole alkaloid; MEP, 2 C-methyl-D-erythritol-4-phosphate; STR, strictosidine synthases. The multiple step reaction is represented by an arrow with a dotted shaft (Sirikantaramas et al. 2013)

The biosynthetic pathway for CPT includes three steps: the pre-strictosidine pathway, strictosidine synthesis, and post-strictosidine pathway. Over the past few decades, numerous biochemical investigations have been carried out in CPT for its enhanced production due to its potential anticancer activity. A metabolic engineering approach is currently adopted for the enhanced CPT production wherein the intermediates involved in the biosynthetic pathway are targeted. Thus, it is necessary to have an in-depth understanding of the CPT biosynthetic pathway (Gonçalves and Romano 2018). Figure 3 illustrates the biosynthetic pathway of CPT.

### Pre-strictosamide pathway

Tryptophan is initially synthesized from chorismate by the shikimate pathway. The chorismate in the presence of enzyme anthranilate synthase converts into anthranilate, which then combines with 5-phosphoribosyl pyrophosphate to form indole glycerol phosphate. Indole is then formed by the addition of α- subunit of tryptophan synthase (TSA) to indole glycerol phosphate, which is then condensed with β- subunit of tryptophan synthase (TSB) to form tryptophan. The tryptophan is decarboxylated to tryptamine by the tryptophan decarboxylase enzyme. Parallelly, secologanin is synthesized from IPP (isopentenyl diphosphate) and its isomer DMAPP (dimethylallyl diphosphate) both of which are intermediates of the 2 C-methyl-D-erythritol-4-phosphate (MEP) pathway and MVA (mevalonate) pathway. IPP and DMAPP condense to form geranyl diphosphate (GPP), which is then converted to geraniol by geraniol synthase. Geraniol is converted to 10-hydroxygeraniol by geraniol 10-hydroxylase and further converted to loganin. Secologanin is synthesized from loganin by secologanin synthase (SLS) (Sirikantaramas et al. 2013).

### Strictosidine synthesis

The tryptamine and secologanin condenses to form strictosidine. This Pictet-Spengler condensation between tryptamine and secologanin is catalyzed by strictosidine synthases (STR) ((Yamazaki et al. 2003).

### Post-strictosidine pathway

In this pathway, CPT is synthesized in a multistep reaction. The strictosidine is converted to strictosamide by undergoing intramolecular cyclization. The strictosamide is converted into pulmioside and deoxypulmioside, ultimately forming camptothecin. The conversion of strictosamide to camptothecin involves oxidation and recyclization of the B and C rings, further oxidation of the D ring and removal of C-21 glucose moiety and final oxidation of the E ring forming camptothecin (Sirikantaramas et al. 2013).

### Production of CPT through plant cell, tissue, and organ culture

**Fig. 4:**
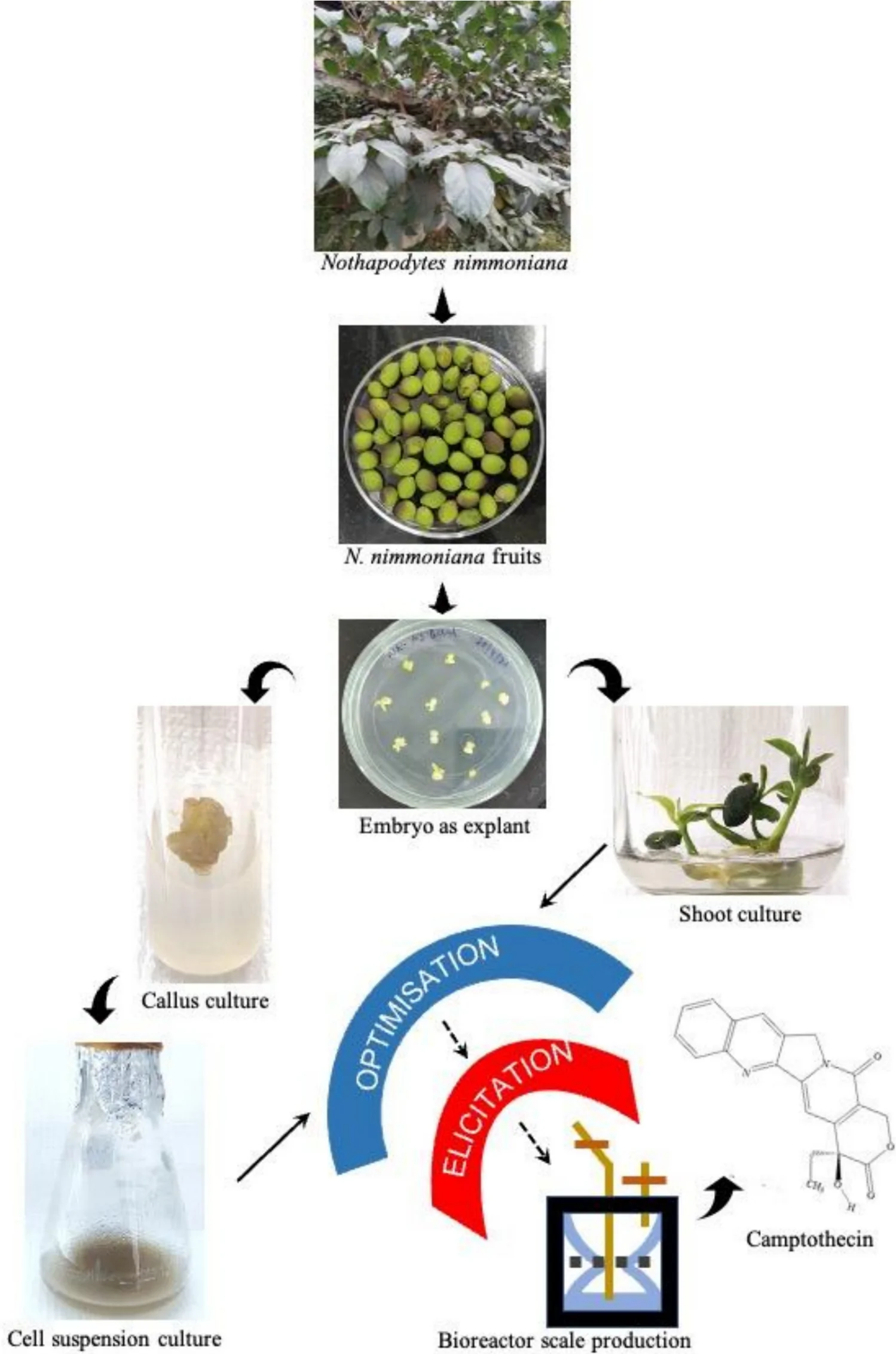
Production of CPT from various tissue culture techniques

Plant tissue culture methods serve as the appropriate alternatives for CPT production to overcome the reduction in the natural population of the plants due to overharvesting. Plant tissue culture techniques involve mass propagation of plants from any part of the mother plant under in vitro conditions without seasonal constraints throughout the year. Moreover, these techniques help in easier isolation and purification of desired secondary metabolites. The secondary metabolites can be produced from an undifferentiated mass of cells, calli, cell suspension cultures and from differentiated shoots, roots (adventitious and hairy roots), or somatic embryos and such plant tissue culture strategies have been adopted for enhancement of camptothecin production (Gonçalves and Romano 2018). Figure 4 demonstrates the production of camptothecin by various tissue culture methods.

### Production of CPT through callus culture and selection of cell lines

The in vitro production of secondary metabolites occurs in two phases: biomass accumulation and secondary metabolite biosynthesis. Both these phases need to be optimized by utilizing a variety of tissue culture strategies (Chandran et al. 2020). Selection of high secondary metabolite yielding cultivars and screening the high secondary metabolite yielding cell lines are the key strategies for increasing secondary metabolite production. The explants are chosen from the high secondary metabolite yielding genotype of the plant. The callus is induced in such explants by exogenous application of auxin or cytokinin or in a defined ratio. The secondary metabolites can be obtained from callus cultured under optimal culture conditions. Furthermore, the callus can be subjected to cell suspension culture for developing fast-growing, high-yielding cell lines (Schreiner 2005).

The explant may exhibit a wide range of metabolic productivity. The heterogeneity would result in decreased secondary metabolite production. These undesirable variations in the production can be avoided or reduced by selecting potentially high-yielding cell populations from heterogeneous cultures. The cloning of such high-yielding cell lines helps in enhanced secondary metabolite production (Smetanska 2008). The effect of different concentrations and combinations of kinetin (KN) and auxin ranging from 0.1 to 10 mg L^−1^ on callus induction of *C*. *acuminata* has been evaluated and reported by Van Hengel et al. (1994). The highest callus biomass was obtained in the MS (Murashige and Skoog medium, 1962) medium supplemented with B5 vitamins, 0.5 mg L^−1^ 2,4-Dichlorophenoxyacetic acid (2,4-D) and 0.1 mg L^−1^ KN with a CPT production of 0.000098% (van Hengel et al. 1992). The cell suspension culture of *Ophiorrhiza eriantha* Wight was established from 16 g friable callus cultured on MS medium with 3% sucrose and 4 mg L^−1^ indole-3-butyric acid (IBA) showed the highest CPT production of 0.087% (Rani 2011). Induction of callus from hypocotyl explant was favorable when compared to leaf explant. It was observed that 2 mg L^−1^ TDZ along with 0.5 mg L^−1^ 2,4-D was favorable for induction of callus without further organogenesis. Callus induced on 2 mg L^−1^ TDZ along with 0.5 mg L^−1^ 2,4-D medium showed two-fold increase when compared to that of callus induced on 1mg L^−1^ TDZ along with 0.5 mg L^−1^ 2,4-D (Kadam et al. 2023). Table 3 illustrates the CPT production by callus culture and cell suspension cultures of various CPT-producing plants.

**Table 3:** Production of CPT through callus culture and cell suspension culture

Plant name	Explant source	Best plant growth regulator (PGR) concentration and combination	Content of CPT	Reference
*C. acuminata*	6-week-old callus induced from stem	B5 vitamins + 0.5 mg L^−1^ 2,4-D + 0.1 mg L^−1^ KN. CAS 3 (Cellosaurus) in MS media + NAA	0.98 mg L^−1^	(van Hengel et al. 1992)
*N. nimmoniana*	Seeds	2 mg L^−1^ Picloram (Pic) + 3% sucrose	9.5 µg g^−1^	(Ciddi and Shuler 2000)
	Mature and immature fruits	4.52 µM 2,4D + 2.22 µM BAP (6-Benzylaminopurine)	1.30%	(Thengane et al. 2003)
	Callus induced from leaf and stem		29.8 µg g^−1^	(Karwasara and Dixit 2013)
		T3 yielded more CPT		(Isah 2017)
*C. grandiflora*	Seed, root, embryos, cotyledon, leaves, hypocotyls	0.5 mg L^−1^ BAP + 3 mg L^−1^ NAA (1-Napthaleneacetic acid)	0.0007 mg g^−1^	(Isah and Umar 2019)
	Internode	-	0.003 mg g^−1^	(Li et al. 2002)
*E. heyneana*	Leaves, internodes, embryos and young seedlings	MS media + 4.52 pM 2, 4- D	0.000025%	(Kulkarni 2008)
*M. dentata*	Leaves, apical buds, node, internode	1 mg L^−1^ IBA + BAP each	0.003-1.4%	(Ramesha et al. 2013)
*N. nimmoniana*	Hypocotyl	2 mg L^−1^ TDZ + 0.5 mg L^−1^ 2,4-D	2 folds higher	(Kadam et al. 2023)
*O. eriantha*	Callus induced from different plant parts	4 mg L^−1^ NAA + 1 mg L^−1^ BAP for callus induction	0.027 mg g^−1^	(Rani 2011)
		4 mg L^−1^ IBA for cell suspension culture	0.087 mg g^−1^	
*O. mungos*	Leaves and tender stem	MS medium + 1.5 mg L^−1^ NAA + 3mg L^−1^ BAP	0.003%	(Jisha 2006)
	Fruit	MS medium + IAA + BAP + Gibberellic acid (GA) in 2:2:1 ratio	0.018%	(Namdeo et al. 2012) Kusari
	Tender leaves	1.0–2.0 mg L^−1^ NAA + 1.0 mg L^−1^ 2,4-D + 0.5 mg L^−1^ KN	0.08 mg g^−1^	(Deepthi and Satheeshkumar 2016)
	Callus induced from tender leaves	3 mg L^−1^ NAA + 1 mg L^−1^ 2,4-D	0.06 mg g^−1^	(Kusari et al. 2009)
*O. trichocarpa*	Callus induced from leaves	1/4 MS media + 0.5 mg L^−1^ KN + 2.0 mg L^−1^ NAA + 1.0 mg L^−1^ + 2, 4-D for callus induction	0.0086 mg g^−1^	(Varghese 2017)
		2.0 mg L^−1^ NAA + 1.0 mg L^−1^ +2, 4-D + 0.5 mg L^−1^ BAP for cell suspension culture	0.0021 mg g^−1^	
*O. pectinata*	Callus derived from shoot tip explants	1mg L^−1^ KN + 2 mg L^−1^ Pic	10.42 µg g^−1^	(Lekshmi 2011)

### Organ culture for CPT production

Secondary metabolite synthesis is often higher in differentiated tissues and organ cultures, and this has been developed as an alternative method for secondary metabolite production. The organ culture method involves the culture of organs such as root, shoot, and embryo and is the most stable method (Gonçalves and Romano 2018). In medicinal plants, shoot cultures have been established for a higher accumulation of secondary metabolites. Similarly, root cultures also serve as an alternative for root-derived secondary metabolites, which are otherwise difficult to harvest from a root system that exhibits slow growth. On the other hand, hairy root cultures have shown to be a viable method for secondary metabolite synthesis in vitro. The hairy roots can be induced by co-culturing the explant with *Agrobacterium rhizogenes*. The T-DNA from the plasmid of *A. rhizogenes* transferred to host tissue induces hairy root formation on account of auxin synthesis genes coded by bacterial DNA. Thus, it avoids the need for an external supply of auxins (Rao and Ravishankar 2002). In the study conducted by Vineesh et al. (2007), a maximum number of shoots were initiated from the leaf of *O. rugosa*, and the highest amount of CPT (0.039%) in multiple shoots was obtained in media with 3% sucrose, 5 mg L^−1^ BAP, and 0.5 mg L^−1^ NAA (Vineesh et al. 2007). It has been reported that the MS media with IBA induces 4–6 adventitious roots/shoot and the media with IBA or NAA alone or NAA combination with BAP or KN produced the wound in the shoot with *A. rhizogenes* 15834 and cultured on B5 medium with 2% sucrose, 0.2% of gellan gum and 200 mg L^−1^ cefotaxime for 7 weeks (Isah and Mujib 2015b). The highest CPT of 471 µg was obtained in hairy roots of *Ophiorrhiza liukiuensis* Hayata (Asano et al. 2004). Table 4 illustrates the total CPT content produced in root and shoot cultures of various CPT-producing plants.

**Table 4:** Production of CPT through shoot and root culture

Plant name	Explant source	Optimal media composition	CPT content	Reference
*C. acuminata*	Shoot tips or axillary buds	9 shoots per explant on 1.5 mg L^−1^ BAP	0.47 mg g^−1^	(Sankar-Thomas et al. 2008)
	In vitro shoots	7 roots per shoot in MS media + 4.9 µM of IBA.	_-	(Jain and Nessler 1996)
	In vitro shoots		6.965 µg g^−1^ (solid media) and 7.472 µg g^−1^ (liquid media	(Yuan et al. 2008)
*Chonemorpha fragrans* (Moon) Alston	In vitro shoots	7.9 roots per shoot in MS media + 0.49 µM IBA + 11.7 µM AgNO_3_		(Nishitha et al. 2006)
*N. nimmoniana*	Calli from nodal explants	165.9 shoots in MS media + 2 µM BAP	0.2823 mg g^−1^	(Dandin and Murthy 2012)
	Stem, zygotic embryo	4–6 roots/shoot in MS media + IBA. high CPT% in MS media + IBA/ NAA alone or combination of NAA + BAP/ KN	0.12%	(Isah and Mujib 2015b)
*I. coccinea*	In vitro shoots	11 roots in BM media + 20.0 µM NAA		(Lakshmanan et al. 1997)
*O. mungos*	Tender leaves, node	MS media + 1 mg L^−1^ kinetin	0.1197%	(Jisha 2006)
	Young fruits	25 shoots in MS medium + 1:2:1 Pic + TDZ (Thidiazuron) + GA	0.0208%	(Namdeo et al. 2012)
*O. mungos*	Callus	Root biomass of 2.918 g in MS media + l mg L^−1^ GA + 1 mg L^−1^ NAA	0.1196%	(Jisha 2006)
	In vitro shoots	22 roots in MS media + 4:1:2 IBA: BAP: GA	0.0324%	(Namdeo et al. 2012)
*O. rugosa*	Leaf, stem and petiole	76 shoots induced in MS media + 4 mg L^−1^ BA and maximum CPT in MS media + 0.1 mg L^−1^BA + 4 mg L^−1^ NAA	0.039%	(Vineesh et al. 2007); Namdeo et al. 2012)
	In vitro shoots	218 roots and maximum CPT content in MS media with 0.05 mg L^−1^ BA and 2 mg L^−1^ NAA	0.065%	
*O. eriantha*	Whole plant parts	26.08 shoots in MS media + 5 mg L^−1^ BAP	0.0485 mg g^−1^	(Rani 2011)
	Callus	16.41 roots and highest CPT in MS media + 4 mg L^−1^ and MS media + 5 mg L^−1^ NAA respectively	0.0794 mg g^−1^	
*O. mungos* L. var. *angustifolia*	Node	22 shoots/explant in media + 8.88 µM BAP	0.14 mg g^−1^	(Mithun et al. 2017)
	Shoot and leaflet	19.48 roots/shoot in MS media + 4.28 µM NAA		
*O. trichocarpa*	25 days old seed and 20 days shoot bud	58.4 shoots in QS MS media + 0.5 mg L^−1^ BAP + 0.05 mg L^−1^ IAA	0.0426 mg g^−1^	(Varghese 2017)
	In vitro shoots	58 roots in media + 0.5 mg L^−1^ IBA	0.0263 mg g^−1^	
*Ophiorrhiza prostrata* D.Don	In vitro shoot	48.2 roots per shoot in MS media + 10.74 µM NAA + 2.32 µM KN	-	(Shahanaz Beegum and Poulose Martin 2007)
*Ophiorrhiza prostrata* D.Don	Leaves, internode, shoot	MS media + 10.74 µM NAA + 2.32 µM kin	0.16%	(Martin et al. 2008)
*O. japonica*	In vitro shoots	24.8 roots per shoot in MS medium + 0.5 mg dm^−3^ IBA	-	(Kai et al. 2008)

### Optimization strategies employed for improved production of CPT

The increasing market demand for secondary metabolites has led to the implementation of traditional and advanced metabolic strategies for incessant high yield production of secondary metabolites (Hussain et al. 2012). The optimization for secondary metabolites can be done in bioaccumulation stages and secondary metabolite synthesis stages. The important parameters such as pH, temperature, light intensity, carbon, and nitrogen source can be optimized at the bioaccumulation stage, and strategies like elicitation, precursor feeding, and immobilization can be achieved at the secondary metabolite biosynthetic stage (Gonçalves and Romano 2018).

### Optimization of culture conditions

The supply of media with carbon, nitrogen, and phosphate sources at optimum concentration influences the secondary metabolite production. These nutrient sources play an important role in signal transduction, thereby regulating the expression of secondary metabolite genes as well as in biomass accumulation. The physical factors like pH, light intensity, temperature, and agitation speed also influence the biomass and metabolites productivity. Thus, choosing the right culture medium formulation is a vital step (Gonçalves and Romano 2018). The effect of sugar concentration on CPT production in cell suspension culture of *C. acuminata* has been studied using different concentrations (2, 4, 6, 8, and 10%) of sucrose, glucose, and fructose in the source media. The highest camptothecin (0.0029 mg L^−1^) was obtained at 6% sucrose concentration (Kim et al. 1999). The effect of nitrogen source on CPT production was studied in *N. nimmoniana* by supplementing the medium with different concentrations of potassium nitrate and ammonium chloride ranging from 60/0 to 0/60 mM NO_3_^−^/NH_4_^+^ balance. The best combination was found to be 50/1 mM NH_4_^+^/NO_3_^−^ balance, with 0.5 mM phosphate on day 15 yielding 0.00474% CPT (Karwasara and Dixit 2013). The cell suspension culture of *C. acuminata* was subjected to pH varying from 4 to 7.5. It was also subjected to two different temperatures 26 and 30 °C with shaking rates of 148 and 184 rpm. The highest biomass was achieved at pH of 4.5, at 30 °C and 184 rpm (Sakato and Misawa 1974). The callus of *C. acuminata* when subjected to different light intensities of 400 W dysprosium lamps ranging from 0 to 100% irradiance, it was observed that highest CPT production of 3.56 mg g^−1^ was achieved at 50% light intensity (Hu et al. 2016). Table 5 tabulates the different optimization strategies that have been adopted for enhanced CPT production.

**Table 5:** Optimization strategies for enhanced camptothecin production

Plant name	Optimal concentration	CPT content	Reference
*C. acuminata*.	6% sucrose	0.0029 mg L^−1^	(Kim et al. 1999)
	40 mM nitrogen with 5:1 NH_4_^+^/NO_3_^−^	6.3 mg L^−1^	(Pan et al. 2004)
	0.2 mM tryptophan pH 4.3 30 °C 184 rpm		(Sakato and Misawa 1974)
	50% irradiance (400 W dysprosium lamps)	3.56 mg g^−1^	(Hu et al. 2016)
	Green light	45.6 µg g^−1^	(Park et al. 2003)
*N. nimmoniana*	5.0% sucrose	47.4 µg g^−1^	(Karwasara and Dixit 2013)
	5:1 NH_4_/NO_3_ with 60 mM total nitrogen	48.7 µg g^−1^	
	0.5 mM phosphate	31.6 µg g^−1^	
	50/1 mM NH_4_^+^/NO_3_^−^, with 0.5 mM phosphate	51.7 µg g^−1^	
*O. rugosa*	3% sucrose	0.558mg g^−1^	(Vineesh et al. 2007)
*O. eriantha*	1% sucrose	0.0679 mg g^−1^	(Rani 2011)
*O. mungos*	3% sucrose	0.002%.	(Jisha 2006)
	20% coconut water	0.04%	
	20% ammonium nitrate	0.04%	
*O. pumila*	63 days after transplanting (DAT); Plant factory with artificial light (PFAL)	380 mg m^−2^ y^−1^	(Lee et al. 2022)
*Pyrenacantha volubilis* Hook.	1240 ppm ammonium nitrate	2.19 mg g^−1^	(Sasidharan et al. 2023)
	5% sucrose	3.16 mg g^−1^	

### Elicitation

Elicitation is another strategy that can be adopted to improve camptothecin production. Elicitors mimic biotic/abiotic attacks, thus eliciting defense mechanisms in plants. The elicitors induce gene upregulation in CPT-producing plants which govern a wide range of cellular activities at the molecular and biochemical levels. Elicitors are classified as abiotic or biotic elicitors. The cell wall fragments of bacteria virus, fungus, enzymes, and molecules such as jasmonic acid (JA) and salicylic acid (SA) are biotic elicitors, and inorganic salts, heavy metals, and physical factors like UV radiation are types of abiotic elicitors (Gonçalves and Romano 2018). CPT can be elicited by elicitors like ferulic acid, methyl jasmonate (MeJA), jasmonic acid (JA), gamma irradiation, and yeast extract (YE) (Song and Byun 1998). The cell suspension culture *O. mungos* was amended with different concentrations of YE ranging from 25 to 200 mg L^−1^ and AgNO_3_ ranging from 2.0 to 7.5 µM. The highest CPT yield of 0.8 mg L^−1^ and 0.52 mg L^−1^ was achieved at 50 mg L^−1^ of YE and 2.5 µM of AgNO_3_, respectively (Deepthi and Satheeshkumar 2016). The effect of gamma radiation elicitation on CPT enhancement in callus cultures of *N. nimmoniana* was studied by irradiating with gamma radiation ranging from 5 to 30 Gy. The enhanced production of CPT in the callus culture was irradiated with 20 Gy radiation yielding 0.098% CPT (Fulzele et al. 2015). Recent reports also suggest that supplementation of yeast extract and glycine helped in increased production of camptothecin from callus cultures of *Chonemorpha fragrans* (Ambujakshi et al. 2022). Table 6 shows the different types of elicitation methods for CPT enhancement in plants.

**Table 6:** Elicitation for enhanced camptothecin production

Plant name	Explant	Optimal elicitor conc.	CPT content	Reference
*C. acuminata*	Cell culture	50 µM JA on day 4 after elicitor dosing	7.1 × 10^−5^ mg g^−1^	(Song and Byun 1998)
	Plantlets	MeJa 10 µM	0.25 mg g^−1^	(Pu et al. 2022)
		PEG 5 g/L	0.21 mg g^−1^	
		AAgNO_3_ gNO_3_ 50 µM	0.26 mg g^−1^	
*N. nimmoniana*	Callus	75mg L^−1^ YE	350–400 µg g^−1^	(Isah 2017)
		20 Gy radiation	0.098% CPT	(Fulzele et al. 2015)
		25 mM CaCl_2_	14.7-fold increase in comparison to control	(Isah et al. 2022)
*E. heyneana*	Callus	50 mg L^−1^ and 100 mg L^−1^ fungal elicitor i.e. *A niger*	CPT undetected	(Kulkarni 2008)
*O. mungos*	Cell suspension	50 mg L^−1^ of YE and 2.5 µM of AgNO_3_	0.8 mg L^−1^ and 0.52 mg L^−1^	(Deepthi and Satheeshkumar 2016)
*O. eriantha*		50 µM MeJA, 1 Gy and 10 mg L^−1^ chitosan	0.251, 0.422 and 0.29 mg g^−1^	(Rani 2011)
*O. mungos*	In vitro shoots	l00µM MeJa for 24 h + 2 Gy	0.47 mg g^−1^ and 0.09% CPT	(Jisha 2006)
	In vitro plants	150 µM MeJA + 50 µM SA	0.23% and 0.15%	(Nagesha et al. 2018)
*Ophiorrhoza kuroiwa*	Hairy roots	100 µM MeJA production by 1.3-fold	0.20–0.25 mg g^−1^	(Asano et al. 2004)
*Pyrenacantha volubilis* Hook.	Green root culture	50 ppm yeast extract	5.13 mg g^−1^	(Sasidharan et al. 2023)
*T. atroviride* LY357*	Fungus isolated from *C. acuminata*	0.05 mM MeJA increased CPT by 3.4 and 2.2-fold	197.82 µg L^−1^	(Pu et al. 2013)

* Indicates endophytes isolated from different plant sources

### Precursor feeding

Precursor feeding has been a well-known and widely used method for increasing secondary metabolite production in plant cells. The basic working principle of precursor feeding is that the intermediate compounds involved in the biosynthetic pathway of secondary metabolites have a fair possibility of improving the yield of the final product. Thus, attempts have been made to enhance secondary metabolites by identifying and supplementing the culture media with precursors. The concentration, time of addition, and type of the precursor should be taken into account for precursor feeding (Rao and Ravishankar 2002). CPT production can be enhanced by supplementing precursors such as tryptamine, loganin, and secologanin which are the intermediate compounds involved in biosynthetic pathways (Silvestrini et al. 2002). *E. infrequens*, the endophytic fungus of *N. nimmoniana*, has been treated with different precursors such as tryptophan, tryptamine, citral, geraniol, leucine, and mevalonic acid either alone or in combination with tryptophan. The highest CPT content of 0.000503% was observed in the sabouraud medium with tryptophan and leucine (Amna et al. 2012). The effect of precursors such as tryptamine and secologanine of varying concentrations of 10, 50, and 100 µM in cell suspension culture of *O. eriantha* has been studied. The enhanced CPT production of 0.00914% and 0.00843% DW was achieved at 50 µM concentration of secologanine and tryptamine, respectively (Rani 2011). *Entrophospora infrequens*, a fungus isolated from *N. nimmoniana*, produced the CPT content 0.8−1 mg g^−1^ when fed with combination of two precursors- tryptophan + leucine (Amna et al. 2012). Table S1 shows the different types of precursors used for CPT enhancement in plants.

### Immobilization

Immobilization of plant cells is a new strategy achieved by encapsulating the plant cell within a solid support. This technique involves the use of hydro colloidal gels such as agarose, calcium alginate, carrageenin, gelatin, and polyacrylamide which entraps the plant material within it. Immobilization plays a crucial role in enhanced production of high value secondary metabolites. It makes it possible for a group of cells to work together at the same time and ensures continual production of the desired metabolites without cell washout, thus increasing the cell’s productivity. The low yield of CPT in the culture medium of large-scale bioreactors has been a major concern. Thus, immobilization of plant cells is employed for the increased production of camptothecin in large scale bioreactors. Mamkulathil Devasia et al. (2021) reported the callus of *O. mungos* has been immobilized for continuous production and to achieve high yield of CPT. It was found that the immobilized callus of *O. mungos* produced 420 µg L^−1^ CPT (Mamkulathil Devasia et al. 2021).

### Omics approaches

Omics approaches such as genomics, transcriptomics, proteomics, and metabolomics can be employed to study and understand the biosynthetic pathway of camptothecin production and the reactions of enzymes that take part in the pathway at gene and protein levels. Genomics helps in the identification and characterization of the candidate genes involved in camptothecin biosynthesis by comparing the genomes of camptothecin-producing plants with non-producing ones. Kang et al. (2021) have obtained a high-quality genome assembly of *C. acuminata* using single-molecule real-time long reads technique (Pacific Biosciences (PacBio) Sequel platform and high-throughput chromosome conformation capture (Hi-C), with which they have further investigated the evolution of camptothecin biosynthesis. It was discovered that *C. acuminata* underwent a whole-genome duplication event, resulting in the emergence of genes involved in camptothecin production. Notably, it was observed that *C. acuminata* lacks a specific enzyme called loganic acid O-methyltransferase (LAMT), but instead has two secologanic acid synthases (SLASs) that convert loganic acid to secologanic acid. The functional divergence of the LAMT gene and positive evolution of two SLAS genes, therefore, contributed to *C. acuminata*’s effective production of camptothecin (Kang et al. 2021). Parallely, in *O. pumila*, metabolite profiling revealed that 3α-(S)-strictosidine, rather than 3-(S), 21-(S)-strictosidinic acid, is the exclusive intermediate involved in CPT biosynthesis (Yang et al. 2021).

In another study by Natarajan et al. (2023), the genomic DNA of *Alternaria burnsii* NCIM 1409 was isolated and sequenced on an Illumina NextSeq500, while the RNA was isolated and the transcriptome analysis was performed with RNA seq. The genome assembly and annotation revealed the presence of candidate genes involved in camptothecin biosynthesis. Comparative genomics analyses with related fungi were further conducted. The study concluded that there was no evidence of horizontal gene transfer from the host plant to the endophyte (Natarajan et al. 2023). In *C. acuminata* treated with elicitors such as MeJa, AgNO_3_, and PEG, 32 genes involved in CPT biosynthesis and 12 CYP450 genes that play a crucial role in the previously unexplored oxidation steps of CPT synthesis were explored (Pu et al. 2022). Genome-wide identification was employed to identify 8 out of 198 APETALA2/ethylene-responsive factor (AP2/ERF) transcription factor genes have been identified to be involved in CPT synthesis regulation with higher level of expression in immature bark and upper stem (Hu et al. 2020). The proteomics and transcriptomic studies in *C. acuminata* have discovered three O-methyltransferases and five cytochrome P450s that involve in camptothecin biosynthesis and 15 transcription factors that regulate CPT biosynthesis (Zhang et al. 2023).

### Metabolic engineering of CPT biosynthesis

Biosynthesis and biotechnological production of CPT have made much progress in recent times. Metabolic engineering is one such approach that has enhanced CPT production. It is a biotechnological discipline that deals with the manipulation of the genes that code enzymes which take part in the biosynthetic pathways. The biosynthetic genes involved in the synthesis of strictosidinic acid and CPT-derivatives have been partially resolved and identified. These genes include CaG10H, Ca10HGO, CaIS, CaSLAS, CaTDC, CaSTRAS, and Ca10OMT. Understanding the functions of these genes allows researchers to precisely manipulate the biosynthetic pathway and enhance CPT production (Fan et al. 2022). Through metabolic engineering, CPT production can be enhanced either by overexpressing the genes that encode the enzymes that are involved in the biosynthesis of CPT or by inhibiting the competitive pathways in turn enhancing the metabolic flux of targeted biosynthetic pathways. Several metabolic engineering studies have been conducted for CPT enhancement in the past decade.

In the study by Cui et al. (2015), the co-overexpression of strictosidine synthase (STR) and geraniol 10-hydroxylase (G10H) genes from *C. roseus* introduced in *O. pumila* yielded 1.77 mg g^−1^ of CPT, i.e., 56% increase (Cui et al. 2015). Furthermore, study by Van der Fits and Memelink (2000) showed that overexpression of *ORCA3* (Octadecanoid-derivative Responsive Catharanthus AP2-domain) from *C. roseus* in *C. acuminata* hairy roots enhance CPT production by 1.5-fold compared by up-regulating the expression key genes involved in terpenoid indole alkaloid (TIA) biosynthetic pathway (van der Fits and Memelink 2000). Metabolic engineering of *CrORCA3* genes in *C acuminata* yielded 0.112% of CPT (Ni et al. 2011). The *OpWRKY2* gene, *OpSLS* (secologanin synthase), *OpG10H*, *STR* genes of *O. pumila*, and *CrG10H* (genes from *Catharanthus roseus* inserted in *O. pumila*) on overexpression yielded 0.00248% (Hao et al. 2021), 0.328%, 0.240% (Shi et al. 2020), 0.177, and 0.128%, respectively (Cui et al. 2015). Furthermore, the overexpression of the NfSTR gene of *O. rugosa* yielded 0.213% of CPT (Singh et al. 2020). In *O. pumila*, it has been discovered that the transcription factor OpWRKY6 plays a key role in regulating camptothecin biosynthesis. Overexpression of OpWRKY6 reduced camptothecin levels, while its knockout resulted in increased camptothecin production, providing valuable insights for enhancing camptothecin production in plants (Wang et al. 2022). In *O. pumila*, the knockout of OpLAMT1 expression led to camptothecin expression, and further OpNAC1 (NAC transcription factor) was demonstrated to suppress the expression of OpLAMT1 and identified as a candidate gene for CPT production (Hao et al. 2023).

### Extraction and quantification of camptothecin

#### Extraction

The extraction method is one of the most imperative methods for the recovery of CPT from its source. It plays a crucial role in the estimation of CPT obtained from various sources. For the extraction of CPT, the development of simple, faster, accurate extraction methods that require a lesser amount of solvents is important. Various extraction methods such as stirring extraction, soxhlet extraction, and microwave extraction have been developed for the extraction of camptothecin (Fulzele and Satdive 2005). Sonication is one of the most commonly used extraction methods. Camptothecin ranging from 0.85 to 3.6% and 0.15 to 0.23% has been extracted by sonication method from callus cultures of *C*. *acuminata* (van Hengel et al. 1994) using 18 mL water and 20 mL dichloromethane solvent, and from *O. mungos* (Nagesha et al. 2018) using 50 mL of methanol, respectively. 0.014% and 0.008–0.0096% of CPT were extracted from cultures of *O. mungos* (Krishnan et al. 2018) and *rugosa* var. *decumbens* (Roja 2006) using methanol solvent by soxhlet extraction method, respectively. 0.51% of CPT has been extracted from *I. coccinea* by water bath extraction method using 61% of methanol by incubating for 3 h at 45 °C (Saravanan and Boopalan 2011). The cultured endophytes have been subjected to different extraction methods such as solvent extraction, water bath extraction, and ultrasonication. The highest CPT content of 283 mg L^−1^ has been extracted by ultrasound-assisted extraction from *F. oxysporum kolhapuriensis* isolated from *N. nimmoniana* (Bhalkar et al. 2015). Table S2 illustrates the different extraction methods for CPT from different plant cultures and endophytes.

### Quantification methods

Quantification of CPT in different plant sources using advanced techniques provides a viable option to determine the possible accurate amounts of CPT production in the plant species, microbial cultures, and in vitro cells. Among these, HPLC is one of the most commonly used methods for CPT estimation. Studies have been conducted on quantification of CPT by HPTLC and TLC methods (Lokesh et al. 2014; Hashim et al. 2016). The CPT content of 0.85–3.6% and 0.70–2.62% was quantified using HPLC-Waters model 510 with acetonitrile: water (25:75) solvent system at 1 mL min^−1^ flow rate (van Hengel et al. 1994), and 0.024–0.030% of CPT has been quantified by using HPLC Jasco PU 2080 in *C. acuminata* (Namdeo and Sharma 2012). About 0.010–0.084% of CPT has been quantified in *N. nimmoniana* using TLC plate coated with silica gel 60F254 pre-coated (20 × 20 cm) using chloroform: ethyl acetate (1: 1) as solvent system (Lokesh et al. 2014). Recently, a new RP HPLC method (90:10; Acetonitrile: Water as the mobile phase, 1 mL/min flow rate at 30 °C) has been developed for estimation of camptothecin in mixed micelles (CPT, PF108, and TPGS). The recovery of camptothecin was determined to be between 98 and 102%, showing that the method proposed is reliable (Patil et al. 2022). Table S2 illustrates the different quantification methods of CPT in different plant cultures and endophytes.

#### Bioreactor studies for the production of CPT

The tremendous market demand requires increased production of CPT. Owing to this, efforts have been made to study the potentiality of its production at a large scale. Thus, the adoption of bioreactor technology has served to enhance CPT production. However, it is one of the most challenging possibilities because of the unstable productivity, slow growth rate, high shear sensitivity, and low oxygen requirement in the plant cells. Bioreactors are highly efficient, predictable, and enable the easy harvest of metabolites from biomass or cultivation media. At present, bioreactors are specially designed for plant tissue cultures which are different from conventional bioreactors. The high shear-stress-sensitive cells are cultured in wave reactors, slug bubble reactors, and undertow reactors, while less shear-sensitive cells are cultured in airlift bioreactors. The optimization of culture conditions and the measurement of biomass productions are the factors that influence secondary metabolite production in these bioreactors (Gonçalves and Romano 2018). About 16.5% camptothecin has been produced on a large scale from hairy roots culture of *C. acuminata* in a 3 L bioreactor (length 235 mm; diameter, 140 mm) with 5-mm stainless steel mesh at 25 °C; 0.25/min aeration rate (Sudo et al. 2002). 0.0045% of CPT has been produced from *Nodulisporium* isolated from *N. nimmoniana* cultured in the bioreactor with 18 L working volume, maintained at an aeration rate of 1 vvm, 0.2 kg/cm pressure, 28 °C, and an agitation rate of 220 rpm (Rehman et al. 2009). Table S3 illustrates the bioreactor studies for large-scale production of CPT by plants and endophytes.

#### Conclusion and prospects

The major sources of camptothecin are the plants, for which a wide variety of valuable plants have been overexploited to meet the increased market demand, and that has resulted in their depletion. Clonal propagation does not yield satisfactory amounts of CPT. Thus, plant tissue culture techniques are the alternative strategies for the sustainable production of CPT rather than overharvesting of the plants, and these techniques serve as an alternative system for sustainable and economical production of camptothecin throughout the year irrespective of climatic conditions. The current review has made attempts to investigate the various plants which are the sources for CPT alkaloid, various endophytes that can serve as a new source for CPT production, plant tissue culture strategies, and the optimization of the culture conditions for both the plants and endophytes. During this exploration, it is evident that there is a crucial need to develop novel techniques for plant tissue culture and refined extraction techniques to increase the production and extraction of such metabolites that are produced in minute quantities in plant parts. Currently, CPT production can be enhanced by using bioreactors in addition to the use of elicitors and precursors. However, in some cases, CPT is produced at very low yields due to limited information about the biosynthetic pathways and the enzymes and genes involved in these pathways. Hence, identifying and understanding the functions of candidate genes involved in the biosynthetic pathways and their engineering using modern biotechnological approaches provides a viable option to enhance CPT production. CRISPR/Cas9 system can be used in the manipulation of the genes that control overexpression of enzymes involved in biosynthetic pathways of CPT and the knockout of genes that are involved in competing pathways.

## Electronic supplementary material

Below is the link to the electronic supplementary material.

Supplementary Material 1
