# Supplementary material for: Biotechnological approaches for the production of camptothecin
Source: Appl Microbiol Biotechnol. 2024 Jun 19;108(1):382. doi: 10.1007/s00253-024-13187-2 (PMC11186875; doi:10.1007/s00253-024-13187-2)
Supplement: Supplementary file 1 — Supplementary Material 1 [file 253_2024_13187_MOESM1_ESM.pdf]

Applied Microbiology and Biotechnology

Biotechnological approaches for the production of camptothecin

Akshatha Banadka[1], Sudheer Wudali Narasimha[1], Vijayalaxmi S Dandin[2], Poornanand M. Naik[3], Amaranatha Reddy Vennapusa[4*], Kalpalatha Melmaiee[4], Ramu S. Vemanna[5], Jameel M. Al-Khayri[6], Muthu Thiruvengadam[7], Praveen Nagella[1*]

[1]Department of Life Sciences, CHRIST (Deemed to be University), Bangalore – 560 029, Karnataka, India.

[2]Department of Biology, JSS College, Dharwad, India.

[3]Department of Botany, Karnatak University, Dharwad 580003, Karnataka, India.

[4]Department of Agriculture and Natural Resources, Delaware State University, Dover, DE 19901, USA.

[5]Laboratory of Plant Functional Genomics, Regional Center for Biotechnology, Faridabad, Haryana 121001, India.

[6]Department of Agricultural Biotechnology, College of Agriculture and Food Sciences, King Faisal University, Al-Ahsa 31982, Saudi Arabia

[7]Department of Crop Science, College of Sanghuh Life Science, Konkuk University, Seoul, South Korea

*Corresponding author: praveen.n@christuniversity.in; Tel: +91-80-40129717; avennapusa@desu.edu; Phone: +17852361351

Table S1 Precursor feeding for enhanced camptothecin production

Plant/ fungus name	Explant	Optimal precursor concentration	CPT content	Reference
C. acuminata	Cell lines	1 mM strictosidine	CPT undetected	(Silvestrini et al. 2002)
E. heyneana	Leaf, callus culture	tryptophan	CPT undetected	(Kulkarni 2008)
O. eriantha	Cell suspension culture	50 µM secologanine + tryptamine	0.0914 mg g^{-1} and 0.0843 mg g^{-1}	(Rani 2011)

Table S2 Various methods for extraction and quantification of CPT from plants and endophytes

**Plant/ Endophyte name**	**Extraction method**	**Quantification method**	**CPT Content**	**Reference**
C. acuminata Decne.	Maceration +rotary evaporation	HPLC (HP 1090)	0.042-0.051%	(Liu and Adams 1996)
	Sonication + Stirring	HPLC (Waters model 510) TLC	0.85-3.6%	(van Hengel et al. 1994)
	Assisted solvent extraction	HPLC (HP1100)	0.048-0.554%	(Li et al. 2002)
	Sonication + vigorous stirring	HPLC (Spectra System)	0.0029 mg L^{-1}	(Kim et al. 1999)
N. nimmoniana	Sonication	-	0.000025%	(Fulzele et al. 2001)
	Microwave-assisted extraction	HPTLC (CAMAG)	350-400 µg g^{-1}	(Isah 2017)
	Sonication	HPLC (Schimadzu)	29.8 µg g^{-1}	(Karwasara and Dixit 2013)

	Maceration		0.1875 g 100 g^{-1}	(Upadhya et al. 2014)
	Maceration	HPLC (Shimadzu SPD-20A)	0.075-0.7%	(Isah and Mujib 2015a)
	Water bath	-	50- 749 µg g^{-1}	(Mithun et al. 2018)
	Solvent	HPLC (Thermo scientific)	0.01%	(Mithun et al. 2017)
	Soxhlet	TLC (E-Merck 60F254)	0.010 % - 0.084 %	(Lokesh et al. 2014)
	Solvent	HPLC (Waters delta-pak)	9.5 µg g^{-1}	(Ciddi and Shuler 2000)
	Sonication		0.70%- 2.62%	(Namdeo et al. 2012)
	Sonication	HPLC (Waters 600 system)	0.0537-0.1555%	(Chang et al. 2014)
E. heyneana	Solvent	-	0.00013%	(Gunasekera et al. 1979)

Tabernaemontana alternifolia L.	Cold	TLC (E-Merck 60F254), HPTLC (CAMAG), HPLC (Perkin Elmer series 2000)	0.0003% - 0.0013%	(Kulkarni et al. 2010)
M. dentata	Water bath	HPLC, LCMS (Shimadzu)	1.0–1.4%	(Ramesha et al. 2013)
M. megacarpum	Solvent + Column chromatography	-	0.053%	(Arisawa et al. 1981)
Pyrenacantha klaineana Pierre ex Exell & Mendonça	Water bath	HPLC, LCMS (Shimadzu)	0.488%	(Ramesha et al. 2013)
I. coccinea	Water bath	HPLC (Shimadzu)	5.0611 µg g^{-1}	(Saravanan and Boopalan 2011)
Ophiorrhiza species	Soxhlet	HPTLC(CAMAG)	0.05 - 476.89 µg g^{-1}	(Rajan et al. 2013)
O. rugosa	Soxhlet	TLC and HPLC (JASCO)	0.0002- 0.00485 %	(Jaimsha Rani et al. 2010)
	Solvent		0.008- 0.0096 %	(Roja 2006)
			0.002- 0.090%	(Roja 2008)
O. alata	Ultrasonication	HPLC (Zorbax Eclipse XDB)	83-785 µg g^{-1}	(Ya-ut et al. 2011)

O. eriantha	Soxhlet	TLC and HPLC (JASCO)	0.002- 0.0485 mg g^{-1}	(Jaimsha Rani et al. 2010)
O. trichocarpa	Soxhlet	HPLC (Gilson 321 series)	0.0021-0.0486 mg g^{-1}	(Varghese 2017)
Ophiorrhiza mungos L.	Water bath	HPLC (Schimadzu)	0.15%- 0.23%	(Nagesha et al. 2018)
Ophiorrhiza mungos var. *Angustifolia*	Solvent	HPLC (Shimadzu)	297.94 µg g^{-1}	(Krishna Kumar et al. 2018)
	Soxhlet		0.14 mg g^{-1}	(Krishnan et al. 2018)
	Sonication	HPLC Jasco 900)	0.0026 -0.0768%	(Namdeo et al. 2012)
O. pumila	Solvent	HPLC	0.03-0.1%	(Saito et al. 2001)
Ophiorrhiza pectinata Arn.	Solvent	TLC(Sisco)	0.001042- 0.00152 %	(Lekshmi 2011)
Ophiorrhiza prostrata D. Don	Solvent	HPLC (Shimadzu)	0.16%	(Martin et al. 2008)
O. kuroiwa	Sonication	HPLC	219.3 µg g^{-1}	(Asano et al. 2004)
I. coccinea	Water bath	HPLC (Shimadzu)	5.0611 µg g^{-1}	(Saravanan and Boopalan 2011))

C. grandiflora	Cold treatment	TLC (E-Merck 60F254)	0.0007-0.0013%	(Kulkarni et al. 2010)
	Solvent	HPTLC (CAMAG)	0.024-0.030%	(Kedari and Malpathak 2014)
*Trichoderma atroviride LY357**	Solvent	HPLC-DAD-HRMS (SSI 1500 series)	197.82 µg L^{-1}	(Pu et al. 2013))
*Aspergillus sp. LY341 and LY355**			7.93 - 42.92µg L^{-1}	
Aspergillus terreus ON908494.1	Solvent	TLC (E-Merck 60F254)	170.5 µg L^{-1}	(El-Sayed et al. 2023)
			150 µg L^{-1}	(El-Sayed et al. 2022)
*Fomitopsis sp. (MTCC 10177) *, Alternaria alternata(MTCC 5477)* and Phomposis sp**	Water bath	LC-MS with HPLC (Dionex Ultimate 3000)	37- 53 µg 100 g^{-1}	(Shweta et al. 2010)
*Fusarium solani**				
	Ultrasonication		0.8mg L^{-1} and 0.52 mg L^{-1}	(Kusari et al. 2009)
*Fusarium oxysporum kolhapuriensis**	Ultra-sound assisted extraction	HPTLC (CAMAG) HPLC (Shimadzu) LCMS Model-	283 mg L^{-1}	(Bhalkar et al. 2015)

Colletotrichum fructicola SUK1 (F1) and Corynespora cassiicola**		G6540B (Agilent Technologies)	146 mg L^{-1}	(Bhalkar et al. 2016)
Entrophospora infrequens, Nodulisporium sp**	Solvent	TLC (64271 Darmstadt Merck K GaA)	0.575 ± 0.031 mg 100 g^{-1}	(Amna et al. 2006)
*Neurospora crassa**	Solvent	HPLC (Luna) LC-MS Bruker-Bremer system	5.5 µg g^{-1}	(Rehman et al. 2008), (Rehman et al. 2009)

* Indicates endophytes isolated from different plant sources

Table S3 Bioreactor studies in plants and endophytes

Plant/ Endophyte name	Explant/ plant source	Bioreactor studies	CPT content	Reference
C. acuminata	Callus	5 L airlift bioreactor,25 °C, 0.33 L min^{-1} aeration, pH 5.8	3.47% ICPTA (isocamptothecin A) 1.23% ICPTB (isocamptothecin B)	(Yu et al. 2005)
O. pumila	Hairy roots	3 L Bioreactor, 25°C; 0.25 min^{-1} aeration rate; 60 rpm agitation rate	0.0023%	(Sudo et al. 2002)
*Entrophospora infrequens **	*N. nimmoniana*	5 L and 18 L batch bioreactor, 28°C, pH 5.6, 1 vvm aeration rate, 200–220 rpm agitation rate	0.00496%	(Amna et al. 2006)
*Nodulisporium**		40 L bioreactor,18 L working volume, 1 vvm aeration rate, 0.2 kg cm^{-1} pressure, 28°C 220 rpm agitation rate	0.0045%	(Rehman et al. 2009)
*Fusarium oxysporum kolhapuriensis**		Bench-scale upflow column bioreactor, whey concentrate powder, 60% moisture, 2.45× 10^{-6} spores/ g, 30 °C, 7 days. 20 mL h^{-1} feeding rate	0.0128%	(Bhalkar et al. 2016)

* Indicates endophytes isolated from different plant sources
